# Comparison of the effect of bipolar coagulation and fibrillar structured oxidized cellulose on formation epidural fibrosis in rats

**DOI:** 10.3906/sag-2002-162

**Published:** 2021-08-30

**Authors:** Eyüp ÇETİN, Burak EREN, Feyza KARAGÖZ GÜZEY, Azmi TUFAN, Abdurrahim TAŞ, Mustafa ÖRNEK, Bülent UYANIK, Tuğçe ÇAY

**Affiliations:** 1 Department of Neurosurgery, Faculty of Medicine, Van Yüzüncü Yıl University, Van Turkey; 2 Department of Neurosurgery, Health Sciences University Bağcılar Training Research Hospital, İstanbul Turkey; 3 Department of Neurosurgery, Beylikdüzü Kolan Hospital, İstanbul Turkey; 4 Department of Genetic, Health Sciences University Bakırköy Training Research Hospital, İstanbul Turkey; 5 Department of Pathology, Health Sciences University Bağcılar Training Research Hospital, İstanbul Turkey

**Keywords:** Epidural fibrosis, failed back surgery syndrome, bipolar coagulation, fibrillar oxidized cellulose, laminectomy

## Abstract

**Background/aim:**

Epidural fibrosis (EF) is a common cause of failed back surgery syndrome seen after spinal surgeries. The most frequent reason for the formation of EF is accumulated blood and its products in the operation zone. On the development of EF, the effect of bipolar coagulation and fibrillar oxidized cellulose, which are used frequently to control bleeding, was investigated.

**Materials and methods:**

In the study, 45 male Sprague Dawley rats were divided into three groups (control, fibrillar, and bipolar). Lumbar laminectomy was applied to all rats under sterile conditions. In the control group, the epidural area was washed with saline solution. Bleeding was controlled with fibrillar oxidized cellulose in the fibrillar group, with bipolar coagulation in the bipolar group. The area to which laminectomy had been applied was removed as a block 6 weeks later and evaluated histopathologically and genetically in terms of EF development. Fibrosis degree was determined histopathologically by counting fibroblasts using the modified Lubina and EF He grading systems. Interleukin-6 (IL-6), transforming growth factor beta-1 (TGFβ-1), and mRNA levels were measured by the droplet digital polymerase chain reaction method.

**Results:**

The number of epidural fibroblasts, percentage of modified Lubina, amount of IL-6, and He grading rates were significantly lower in the fibrillar group than in the bipolar and control groups (p ˂ 0.05). On the other hand, there was no significant difference among the control, fibrillar, and bipolar groups in terms of TGFβ-1 values (p= 0.525).

**Conclusion:**

The use of fibrillar oxidized cellulose was more effective for hemostasis than bipolar coagulation in reducing the development of EF.

## 1. Introduction

Failed back surgery syndrome (FBSS) is a complication observed after lumbar region surgery, and its rate in patients who undergo lumbar region surgeries is approximately 15% [1]. FBSS is a postoperative condition that does not meet the preoperative expectations of surgeons and patients and affects the patient’s quality of life [2]. One reason for FBSS is epidural fibrosis (EF), which is the development of an intense amount of fibrosis in the epidural area after spinal surgery [3]. Surgical reasons, such as using many cotton pads, extensive pulling of roots, extensive bleeding, and root anomalies, have a role in the etiology of EF [2]. 

Epidural Fibrosis is the replacement of epidural fat with fibrotic tissue. İt consists of connection between the dura mater and another neural structures. İt can cause symptomatic problems, such as radicular pain. İt is showed a close relationship between epidural adhesions and recurrent radicular pain or lower back pain after a lumbar discectomy operation [4].

Furthermore, epidural adhesion leads to various difficulties for revision surgery. The rate of complications, such as iatrogenic nerve root wound or dura mater tear, continues unpredictably high in revision surgeries. Consequently, prevention of epidural fibrosis and adhesion is an imperative for a successful lumbar discectomy [5].

The relationship between systemic rheumatic diseases and IL-6 and TGFβ-1 has been shown in previous studies. This shows that IL-6 and TGFβ-1 levels increase in inflammatory processes [6]. These inflammatory processes that trigger postop fibrosis changes in the epidural area are the activation of the cascade [7].

Many experimental studies have been conducted to prevent EF, and some materials have been shown to be useful. We determined the effectiveness of bipolar coagulation and fibrillar oxidized cellulose on reducing EF formation in rats.

## 2. Material and methods

This study of animal experiments was performed in the Bağcılar Training and Research Hospital between April 2017 and May 2017. Project approval number and date: 2016-39/22.11.2016. One-year-old male Sprague Dawley rats (n = 45; average weight 350 ± 50 g) were used. Rats were divided into three groups (15 rats in each group) and placed in cages with a 12-h light/dark cycle, ambient temperature 20–25 °C, and humidity 60%. Food and water were given ad lib.

Animals were put under general anesthesia by applying ketamine hydrochloride (Ketalar, Pfizer, İstanbul, Turkey) 100 mg/kg intraperitoneal (i.p) and xylazine hydrochloride (Rompun, Bayer, Turkey) 10 mg/kg i.p. rats were placed in the prone position. After the L5 skin level was determined, the skin was shaved, povidone iodine antiseptic solution was applied, and the area was covered as sterilized. The skin and the area under the skin at the median L5 level were incised with a #15 bisturi blade. Bilateral blunt dissection was performed to the prevertebral fascia. The L5 lamina was revealed, and L5 laminectomy was performed to the dura.

In Group 1 (L5 laminectomy [control] group), the dura was washed with saline solution. In Group 2 (L5 laminectomy + bipolar coagulation), venous plexus bleeding on both sides of the spinal cord was controlled using bipolar coagulation. After the bleeding stopped, the surgery zone was washed with saline solution. In Group 3 (L5 laminectomy + fibrillar oxidized cellulose; Ethicon, Son Lorenzo, Puerto Rico), venous plexus bleeding on both sides of the spinal cord was controlled with fibrillar oxidized cellulose. After the bleeding stopped, the surgery zone was washed with saline solution.

In all rats, the skin was closed with 2-0 polyglactine (vicryl) suture (Doğsan, Trabzon, Turkey). The wounds were dressed, and the rats were put in their cages after they woke up. Within the first hour postoperatively, four rats died (one from the fibrillar and three from the bipolar groups). The study continued with the remaining 41 subjects.

### 2.1. Sample collection

Rats were sacrificed 6 weeks postoperatively using high dose ketamine hydrochloride and xylazine hydrochloride i.p. Then, the spine segment (approximately 2 cm thick) consisting of the surgery zone was removed en bloc and divided into two equal parts in the coronal plane so as to leave the laminectomy area at the center. One part was sent for histopathologic examination in 10% buffered formaldehyde. Approximately 1 cm² soft tissue taken from the epidural region of the other piece was placed in the tubes for genetic sampling and stored at −70 °C in liquid nitrogen. Then, those samples were transported to the genetic laboratory with a cold chain.

### 2.2. Histopathologic examination

**Figure 1:**
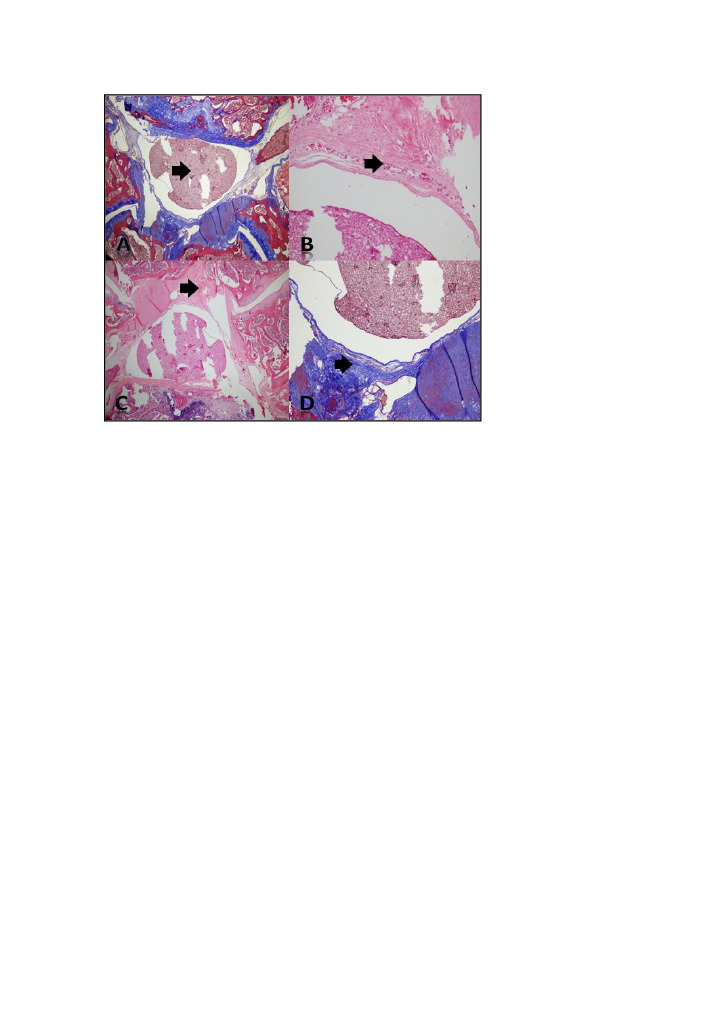
(a) Photomicrographs showing the spinal cord of the groups. Massontrichrome. Black arrow: spinal cord, (b) Fibroblast density, note the increased number of fibroblast cells, Hemotoxylin & Eosin. Black arrow: Fibroblast, (c) Photomicrographs demonstrating epidural fibrosis in the control group, Hemotoxylin & Eosin. Black arrow: fibrosis tissue, (d) Photomicrographs demonstrating epidural fibrosis in the control group, massontrichrome. Black arrow: fibrosis tissue

The spine segment obtained was fixed in 10% buffered formaldehyde for 72 h. Then, it was placed into a “short decalcification solution” and was kept there until the bony tissue softened. After 24-h routine tissue monitoring, tissues were put in paraffin blocks, cut in 4-µm thick sections, and stained with hematoxylin and eosin and Masson’s trichrome to evaluate fibrosis. (Figures 1a and 1b) Sections were evaluated by a light microscope at ×400 magnification. Fibrosis degree was determined histopathologically by counting fibroblasts using the modified Lubina and EF [4]. (Figures 1c and 1d) and He grading system [5].

### 2.3. Genetic examination

To detect interleukin-6 (IL-6) and transforming growth factor beta-1 (TGFβ-1) gene expression differences between groups, the droplet digital polymerase chain reaction (ddPCR) method was used.

For RNA isolation, the classical phenol–chloroform method and PureZOL RNA Isolation Reagent (catalog no: #7326880; Bio-Rad Laboratories, Hercules, CA, USA) kit were used. To create complementary DNA (cDNA) from isolated mRNA copy, the IScript cDNA Synthesis Kit (catalog no: #1708891; Bio-Rad Laboratories) was used. TaqMan Gene Expression Assay (catalog no:4331182; Thermo Fisher Scientific, Waltham, MA, USA) was used for quantitation of obtained cDNA copy with the ddPCR method, catalog no:Rn00572010 m1 (Thermo Fisher Scientific) was used for TGFβ-1, catalog no: #Rn01410330 m1 (Thermo Fisher Scientific) was used for IL-6 and catalog no: Rn01775763g1 (Thermo Fisher Scientific) TaqMan probes were used for glyceraldehyde-3-phosphate dehydrogenase (GAPDH) as a housekeeping gene. Each sample was studied twice. Analysis after the ddPCR process was done using Quanta Soft 1.7 (catalog no:1864011; Bio-Rad Laboratories) software.

TGFβ-1 and IL-6 quantitation values were normalized with GAPDH values for each sample and were averaged for the control, bipolar, and fibrillar groups. The rate of change of mean TGFβ-1 and IL-6 quantitation values for the bipolar and fibrillar study groups was compared according to values determined for the control group.

### 2.4. Statistical analysis

Mean, standard deviation, median lowest and highest, frequency, and rate values were used in the descriptive statistics of data. Distribution of the variables was measured by the Kolmogorov–Smirnov test. Kruskal–Wallis and Mann–Whitney U tests were used for the analysis of quantitative independent data, and the χ^2^ test was used for the analysis of qualitative independent data. The SPSS v: 22.0 program (SPSS, Inc., Chicago, IL, USA) was used in the analyses.

## 3. Results

**Figure 2:**
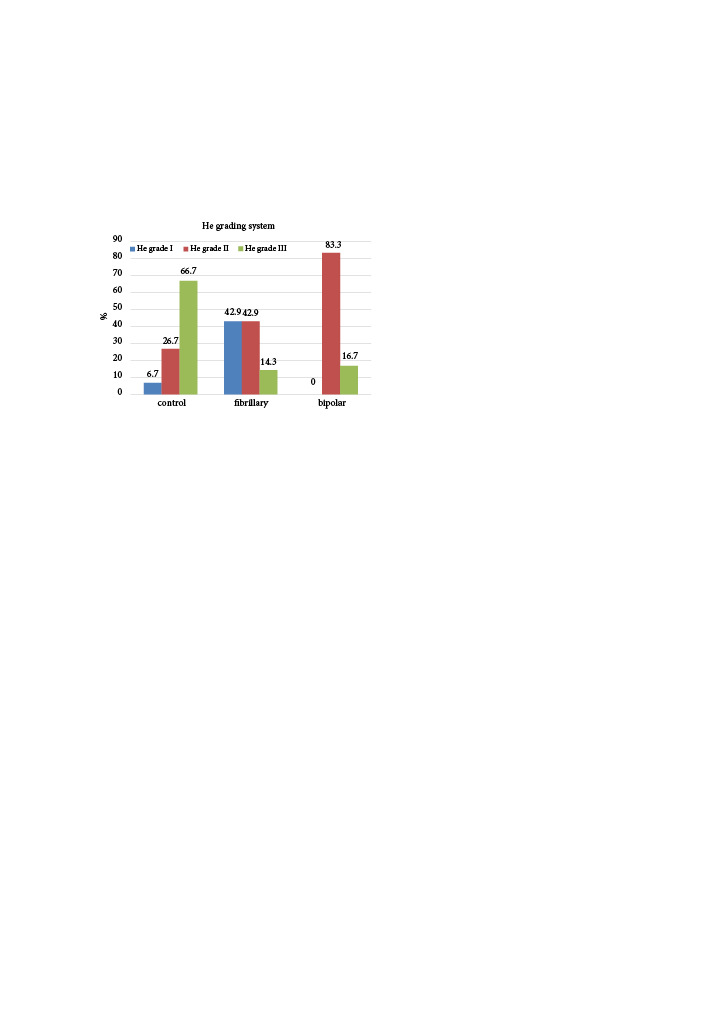
Distribution of the groups according to the epidural fibrosis He grading system. EF was significantly lower in the fibrillar and bipolar groups than in the control group (p = 0.004). There was no significant difference in EF between the fibrillar and bipolar groups (p = 0.867).

According to the He grading system, EF was significantly lower in the fibrillar and bipolar groups than in the control group (p = 0.004) (Figure 2). There was no significant difference in EF between the fibrillar and bipolar groups (p = 0.867).

**Figure 3:**
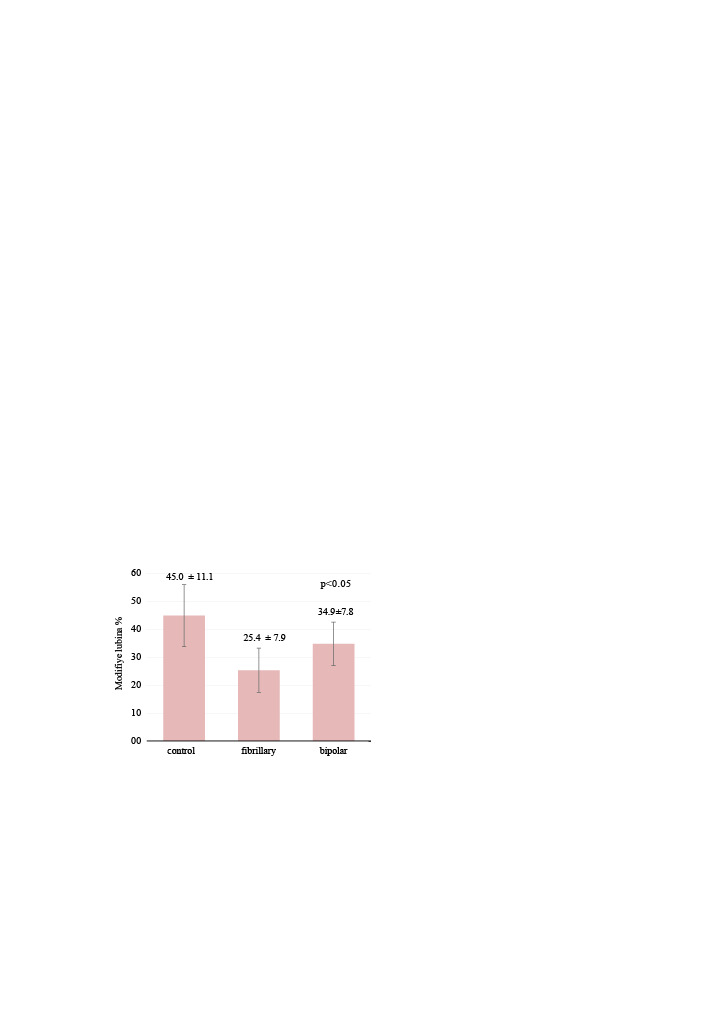
Comparison of the groups according to the modified Lubina system. EF rates were significantly lower in the fibrillar group than in the other groups (p = 0.0001). In addition there was a significant difference in the bipolar group compared with in the control group (p = 0.009).

According to modified Lubina grading, EF rates were significantly lower in the fibrillar group than other groups (p = 0.0001) (Figure 3). In addition, there was a significant difference in the bipolar group compared with the control group (p = 0.009).

**Figure 4:**
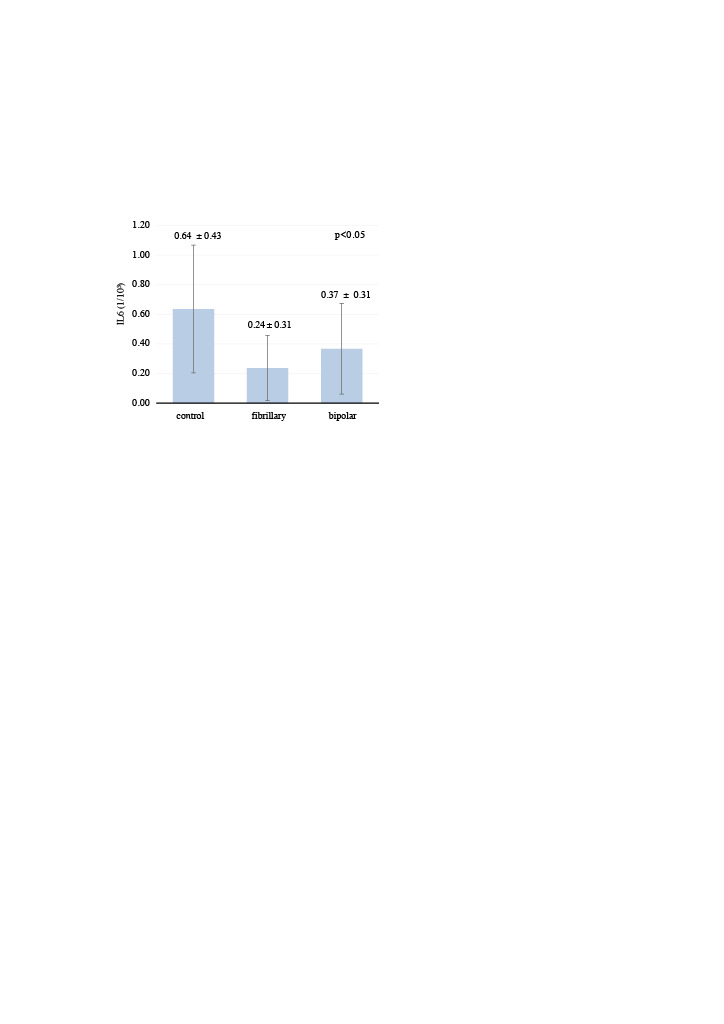
Distribution of IL-6 levels in the groups. In the fibrillar group, the IL-6 (1/10³) level was 0.24 ± 0.31, which was significantly lower than that in the control and bipolar groups (p = 0.022). There was no significant difference in the IL-6 level between the control and bipolar groups (p = 0.142).

**Figure 5:**
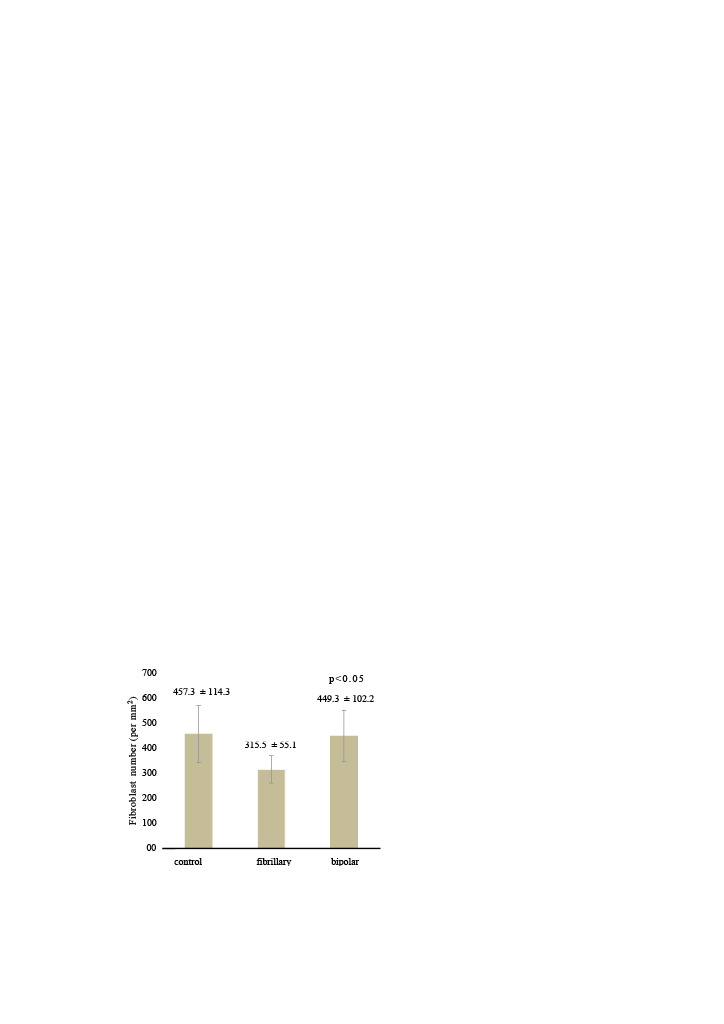
Distribution of fibroblast numbers in the groups. There was no significant difference in the fibroblast number between the control and bipolar groups (p = 0.961). In the fibrillar group, the fibroblast number was 315.5 ± 55.1, which was significantly lower than the levels in the control and bipolar groups (p = 0.001)

**Figure 6:**
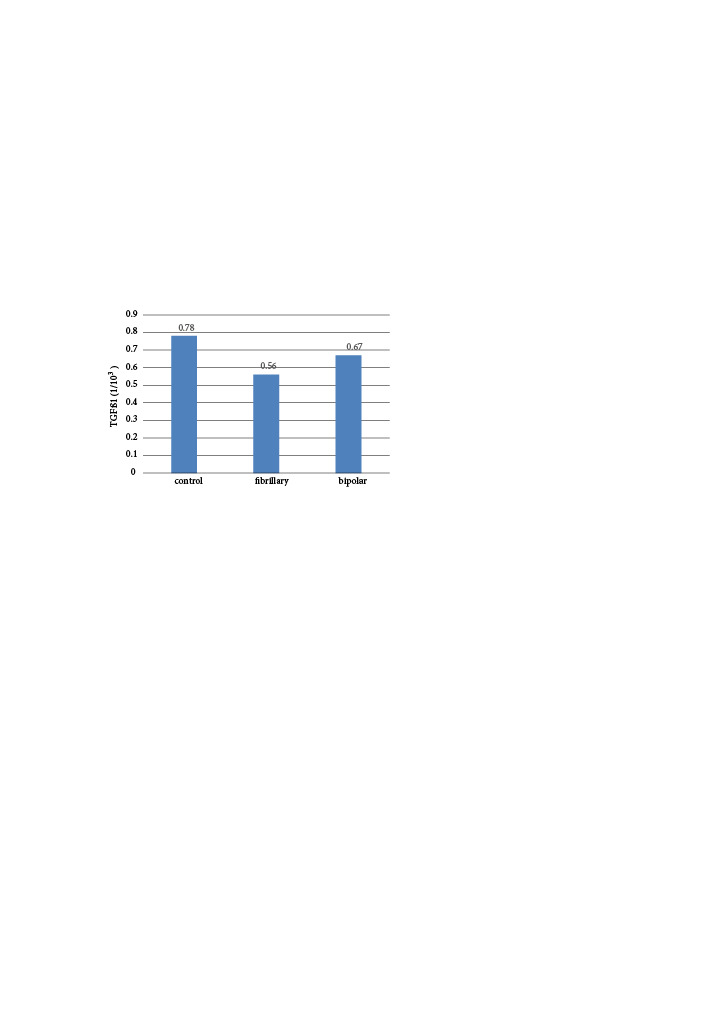
Distribution of TGFβ1 levels in groups. TGFβ-1 levels were 0.69 ± 0.24, 0.58 ± 0.30, and 0.75 ± 0.38 in the control, fibrillar, and bipolar groups, respectively. There was no significant difference among the control, fibrillar, and bipolar groups in terms of TGFβ-1 values (p = 0.525).

In the fibrillar group, the fibroblast number was 315.5 ± 55.1 and IL-6 (1/10³) level was 0.24 ± 0.31, which were significantly lower than those in the control and bipolar groups (p = 0.001 and p = 0.022, respectively; Table 1 and Table 2) (Figure 4). There was no significant difference in fibroblast number (p = 0.961) (Figure 5) or IL-6 level (p = 0.142) between the control and bipolar groups. TGFβ-1 levels were 0.69 ± 0.24, 0.58 ± 0.30, and 0.75 ± 0.38 in the control, fibrillar, and bipolar groups, respectively (p = 0.525, Table 3) (Figure 6).

**Table 1:** Fibroblast count in the rat groups.

Group	Number of fibroblasts			P value
	Min–max	Median	Mean ± SD	
control	323.0–642.0	455.0	457.3 ± 114.3	
fibrillar	231.0–391.0	313.0	315.5 ± 55.1	0.001
bipolar	267.0–609.0	458.0	449.3 ± 102.2	

Kruskal–Wallis (Mann–Whitney U test).

**Table 2:** IL-6 expression in the rat groups.

Group		IL-6(1/103)		P value
	Min–max	Median	Mean ± SD	
control	0.14–1.35	0.47	0.64 ± 0.43	
fibrillar	0.05–1.12	0.10	0.24 ± 0.31	0.022
bipolar	0.08–0.96	0.21	0.37 ± 0.31	

Kruskal – Wallis

**Table 3:** TGF-B1 expression in the rat groups.

Group	TGFB1 (1/103)			P value
	Min–max	Median	Mean ± SD	
control	0.26–0.94	0.78	0.69 ± 0.24	
fibrillar	0.13–1.08	0.56	0.58 ± 0.30	0.525
bipolar	0.25–1.55	0.67	0.75 ± 0.38	

Kruskal–Wallis (Mann–Whitney U test) Note: a = control with fibrillar, p = 0.356, b = fibrillar with bipolar, p = 0.320, c = control with bipolar, p = 0.925.

## 4. Discussion

EF is a common cause of FBSS after spinal surgeries. EF is seen in various degrees after invasive operations applied to the spinal region. EF results from the involvement of fibroblasts caused by traumatized paraspinal muscles in hematoma [7]. While most patients are not clinical, symptoms are seen in a small number of patients. Ross et al. [8] reported that 83% of patients with EF had no symptoms. Symptomatic cases are treated with medical and physical therapy, and surgical intervention is also performed in some cases even if the probability of success is low.

As a result of the effect of cytokines released from blood products left in the surgery zone, production and proliferation of fibroblasts increase and cause EF formation. For this reason, it is thought that the lesser the blood that remains in the epidural area, the lower the risk of EF formation [7]. Therefore, in our study, the effectiveness of bipolar coagulation and fibrillar hemostats, which are most commonly used for bleeding control, were investigated to reduce blood products collected in the operation zone. Fibrillar oxidized cellulose is a woven fiber matrix consisting of water-soluble, oxidized etherized, regenerative cellulose derivatives. Although effect mechanism of fibrillar oxidized cellulose has not been revealed definitely, it has been thought to increase platelet activity on factor 12 and facilitate thrombosis within 2–3 min. Depending on the amount placed and presence of fluid in the area of application, it is completely absorbed by the body at various speeds. It reportedly triggers fibrosis due to its damage to surrounding tissues in the region where bipolar coagulation is applied [9]. This may be due to the inability to clear necrotic tissues from the operation site. However, fibrillar oxidized cellulose is a tissue compatible and absorbable material.

Because there is a relationship between EF grade and ongoing pain after the intervention, several studies have been undertaken to prevent EF formation. Mirzai et al. [10] reported in their prospective randomized study that drain placement in the surgical drastically area reduced hematoma in the area, suggesting that this could prevent postoperative EF formation. Gurer et al. [11] used rosuvastatin, which is used to treat hyperlipidemia, in an experimental study and reported that it significantly reduced EF development. Another experimental study showed that tacrolimus, applied topically, reduced postoperative thickness of the epidural tissue [12]. Karatay et al. [13] reported that bevacizumab, a vascular endothelial growth factor inhibitor used in oncology, drastically reduced EF development in rats after laminectomy. Another experimental study that was conducted using pimecrolimus and mitomycin C, immunosuppressant used for atopic dermatitis, reported similar results [14]. Ozdemir et al. [15] showed that colchicine used as an antipyretic and analgesic for Familial Mediterranean Fever drastically reduced EF. Apart from this, experimental studies have shown that hyperbaric oxygen therapy [16], etanercept (a tumor necrosis factor-alpha inhibitor used to treat rheumatic diseases) [17], ketoprofen (a nonsteroidal antiinflammatory drug) [18], and verapamil (a calcium channel blocker) [19] reduced EF formation after laminectomy.

Studies conducted with hemostats or adhesion inhibitors in the literature reported that EF formation was reduced. Ozay et al. [20] reported that the hemostatic agents, such as ankaferd hemostat and microporous polysaccharide hemosphere, drastically reduced EF development. Similarly, ADCON-L, a carbohydrate polymer, was used in laboratory and clinical studies; this antiadhesion barrier gel significantly reduced EF [21,22]. At present, ADCON-L is used regularly in some clinics based on these results [21]. Gürçay et al. [23] compared the effects of ADCON-L, fibrillar oxidized cellulose, and polysaccharide hemostat on EF development in their study and, as a result, they reported that polysaccharide hemostat and fibrillar oxidized cellulose reduced EF development as much as did ADCON-L. Similarly, we found that fibrillar oxidized hemostat reduced EF development compared to bipolar coagulation, the simplest epidural hemostatic method in spinal surgery.

TGFβ-1 and IL-6 mediators were reportedly important in acute and chronic inflammation, and inflammation increased significantly as a result of the emergence and release of IL-6 [24]. We demonstrated in our study that IL-6 gene expression was significantly low in the fibrillar oxidized cellulose group. There was no difference in TGFβ-1 value between the groups. When evaluated histopathologically, results indicated that the mediator, effective in EF formation, was IL-6. In a study conducted with verapamil, Wang et al. [19] found that IL-6 and TGFβ-1 decreased in parallel with EF, but the decrease in IL-6 level was more prominent.

This experimental study has some limitations. The use of only oxidized cellulose by the surgeon for coagulation is rare because bipolar coagulation has been so widely used and it is effective. If bipolar coagulation is not enough, maybe there is some necessity for using fibrillar oxidized cellulose. We have investigated the efficacy of fibrillar oxidized cellulose, which is easily accessible and used frequently in daily practice, and bipolar coagulation used routinely in all surgeries on EF development. As a result of bipolar coagulation, EF occurred less often, but we found that fibrillar oxidized cellulose significantly reduced EF development as a result of pathological and genetic studies. Our results suggested that fibrillar oxidized cellulose placed in the epidural region for bleeding occurring during spinal surgery is a reliable agent in terms of preventing the development of epidural fibrosis. We anticipate that the use of fibrillar oxidized cellulose in preventing epidural fibrosis will also decrease unsuccessful lumbar surgery.

Back pain that does not stop after spinal surgery is a very difficult situation for patients and physicians and causes many financial, psychological, and social difficulties. Studies have been conducted to reduce EF formation, which is a common reason for the difficulty, and studies are still ongoing. EF rates were significantly lower in the fibrillar group than in the other groups. IL-6 levels were found to be significantly lower in the fibrillar group when compared with the control and bipolar groups. It has been considered that the use of fibrillar oxidized cellulose for bleeding control during surgery would further prevent the formation of epidural fibrosis. However, it should be remembered that the excessive use of this agent during surgery might cause pressure findings in the epidural region and root compression by causing clotting. Many more studies are needed to prevent epidural fibrosis.

## Ethical approval

This experimental study was approved by the animal experiments ethics committee of Bağcılar Training and Research Hospital (Project Number: 487022).

## References

[ref1] (1991). Lumbar disc surgery: results of the prospective lumbar discectomy study of the joint section on disorders of the spine and peripheral nerves of the American Association of Neurological Surgeons and the Congress of Neurological Surgeons. Neurosurgery.

[ref2] (2009). Effectiveness of spinal endoscopic adhesiolysis in post lumbar surgery syndrome: a systematic review. Pain Physician.

[ref3] (2015). Epidural fibrosis after lumbar disc surgery: prevention and outcome evaluation. Asian Spine Journal.

[ref4] (1996). Association between peridural scar and recurrent radicular pain after lumbar discectomy: magnetic resonance evaluation. Neurosurgery.

[ref5] (1991). Causes of failure of surgery in the lumbar spine: 10 year follow-up. The Mount Sinai Journal of Medicine.

[ref6] (1997). McGill PE. British Journal of Rheumatology.

[ref7] (2016). Experimental model of intervertebral disk mediated postoperative epidural fibrosis. Annals of Neurosciences.

[ref8] (1996). Association between peridural scar and recurrent radicular pain after lumbar discectomy: magnetic resonance evaluation. Neurosurgery.

[ref9] (2017). Comparison between resection, bipolar coagulation and Plasmajet: a preliminary animal study. European Journal of Obstetrics & Gynecology and Reproductive Biology.

[ref10] (2006). Are drains useful for lumbar disc surgery?: a prospective, randomized clinical study. Clinical Spine Surgery.

[ref11] (2015). Evaluation of topical application and systemic administration of rosuvastatin in preventing epidural fibrosis in rats. The Spine Journal.

[ref12] (2011). Topical application of tacrolimus prevents epidural fibrosis in a rat postlaminectomy model: histopathological and ultrastructural analysis. Turkish Neurosurgery.

[ref13] (2012). The effect of bevacizumab on spinal epidural fibrosis in a postlaminectomy rat model. Turkish Neurosurgery.

[ref14] (2009). Use of pimecrolimus to prevent epidural fibrosis in a postlaminectomy rat model. Journal of Neurosurgery Spine.

[ref15] (2010). Topical use of colchicine to prevent spinal epidural fibrosis in rats. Neurological Research.

[ref16] (2013). Hyperbaric oxygen in epidural fibrosis: is there a potential for treatment?. Turkish Neurosurgery.

[ref17] (2014). The effect of etanercept on spinal epidural fibrosis in a postlaminectomy rat model. Turkish Neurosurgery.

[ref18] (1995). A quantitative model of post-laminectomy scar formation. Effects of a nonsteroidal anti-inflammatory drug. Spine.

[ref19] (2014). Calcium channel blockers in reduction of epidural fibrosis and dural adhesions in laminectomy rats. European Journal of Orthopaedic Surgery & Traumatology.

[ref20] (2015). The effects of ankaferd blood stopper and microporous polysaccharide hemospheres on epidural fibrosis in rat laminectomy model. Acta Cirurgica Brasileira.

[ref21] (2001). Results of applying ADCON-L gel after lumbar discectomy: the German ADCON-L study. Journal of Neurosurgery Spine.

[ref22] (2017). Is the use of hemostatic matrix (Floseal) and alkylene oxide copolymer (Ostene) safe in spinal laminectomies? Peridural fibrosis assessment. Acta Orthopaedica Et Traumatologica Turcica.

[ref23] (2016). Evaluation of topical application of polysaccharide hemostat and oxidized regenerated cellulose on epidural fibrosis in rats. Journal of Neurological Sciences (Turkish).

[ref24] (2011). The pro-and anti-inflammatory properties of the cytokine interleukin-6. Molecular Cell Research.

